# A Typology of Food Environments in the Pacific Region and Their Relationship to Diet Quality in Solomon Islands

**DOI:** 10.3390/foods10112592

**Published:** 2021-10-27

**Authors:** Jessica R. Bogard, Neil L. Andrew, Penny Farrell, Mario Herrero, Michael K. Sharp, Jillian Tutuo

**Affiliations:** 1 Agriculture and Food, Commonwealth Scientific and Industrial Research Organisation (CSIRO), Brisbane, QLD 4067, Australia; 2 Australian National Centre for Ocean Resources and Security (ANCORS), University of Wollongong, Wollongong, NSW 2522, Australia; nandrew@uow.edu.au (N.L.A.); michaels@spc.int (M.K.S.); 3 Menzies Centre for Health Policy, Sydney School of Public Health, University of Sydney, Sydney, NSW 2006, Australia; penny.farrell@sydney.edu.au; 4 Department of Global Development, College of Agriculture and Life Sciences and Cornell Atkinson Center for Sustainability, Cornell University, Ithaca, NY 14850, USA; mh2258@cornell.edu; 5 Pacific Community, Noumea 98800, New Caledonia; 6 WorldFish, Honiara P.O. Box 438, Solomon Islands; J.Wate@cgiar.org

**Keywords:** food environment, nutrition, Pacific, food system, diet quality, Solomon Islands

## Abstract

Extensive literature describes the importance of food environments (FEs) as a driver of food choices and nutrition outcomes; yet existing FE frameworks do not adequately capture the diversity of FEs relevant to the Pacific Region. This limits identification of opportunities in food systems to reduce the multiple burden of malnutrition. We present a conceptual typology of FEs including six primary FEs relevant in the Pacific; wild; cultivated; kin and community; informal retail; formal retail; and food aid and services. We then apply this typology to food acquisition data from Solomon Islands 2012/13 Household Income and Expenditure Survey and analyse the relationship between FEs and diet quality. The cultivated FE accounts for 60% of the quantity of food acquired nationally, followed by wild (15%), kin and community (9%), and formal and informal retail FEs (8% each), with wide variation between urban and rural households, provinces and wealth groups. Reliance on different FEs is a significant predictor of diet quality and affirms the importance of subsistence fisheries and agriculture, and community and kinship networks. Integration of a FE typology such as the one presented here in commonly conducted household expenditure surveys offers significant opportunity to advance our understanding of food system leverage points to improve nutrition and health.

## 1. Introduction

Malnutrition is ubiquitous throughout the world with hunger and undernutrition affecting one in nine people, and overweight or obesity affecting one in three people globally [1]. This comes at huge health, social and economic costs to countries and communities. Not only are food systems failing to nourish populations around the world, but they are also contributing to unprecedented environmental damage in terms of biodiversity decline, soil degradation, water use, deforestation, and contribution to climate change [2]. The triple challenges of undernutrition, overweight and obesity and climate change have been described as a global syndemic, together posing one of the greatest challenges of the 21st century [3]. The need for food systems transformation to address these global challenges has been called for by numerous high-level meetings [4,5], reports [6,7], targets and policies [8,9].

Food environments (FEs) are a central component of food systems, but are understudied and methods, tools and indicators for FEs are under-developed, particularly in low- and middle-income countries (LMICs) [10,11]. The FE can be conceptualised as including all of the places and pathways through which people acquire and/or consume food, and the various characteristics of those environments that influence food choices [6,12]. Common FEs, particularly in urban settings and high-income countries (HICs), include the built FE such as supermarkets, retailers, restaurants, and convenience stores, which have been the focus of the majority of FE research to date [13]. More recently, cultivated and wild FEs have also been recognised as important sources of food acquisition, particularly in rural settings and LMICs. Wild FEs include the collection of foods produced in environments such as jungles, forests and waterbodies, and cultivated FEs include production systems such as gardens, fields and aquaculture [14]. Characteristics of FEs that may influence food choices include what foods are available, their relative price and affordability, how foods are promoted, their quality, convenience, mode of exchange, sustainability and other factors. FE research originates from social ecological theory which posits that behaviour is determined not only by individual factors, but also sociocultural, structural and environmental factors and policies [15]. A large body of literature examines the role of FEs as a driver of obesity [16,17], emerging largely from HICs. Several recent studies have identified the need for FE research in LMIC settings to understand its role as a driver of the double burden of malnutrition [12,18].

Pacific Island Countries and Territories (PICTs) suffer a disproportionately large double burden of malnutrition among adults and children; 38.2% of children under 5 years are chronically stunted, 9.1% of children under 5 years are overweight, 47% of adolescents are overweight or obese, and around 90% of adults are overweight or obese [19] (figures cited are for the Oceania region which includes Australia, New Zealand and 12 PICTs). The 22 PICTs suffer some of the highest rates of non-communicable diseases (NCDs) globally. Of the 10 countries with the highest prevalence of diabetes globally, seven are PICTs [20]. Pacific food systems are undergoing a transition from traditional diets based on root vegetables and fish sourced from local production systems to increasing dependence on imported foods which are often highly processed and high in saturated fat, added sugar and salt [21,22,23,24].

The FE is a critical leverage point to support healthy and sustainable diets because it ‘contains the total scope of options within which consumers make decisions about which foods to acquire and consume’ [14]. Yet, evidence on *how* to leverage FEs through effective interventions, particularly in the Pacific region is limited by a lack of validated and consistent methodologies and tools to characterise those environments. For example, whilst standardized methodologies are widely used to assess food prices and affordability [25], such methods are designed for formal retail FEs, and not directly transferable to informal market retail or wild and cultivated FEs common in LMICs including many PICTs. Similarly, highly developed geographic analysis is often used to measure availability or convenience of FEs using secondary data sources such as registers of formal food retail businesses and road network data [13]. Such data sources are often unavailable for LMICs, and less relevant in the Pacific region where formal retail FEs are relatively uncommon for the largely rural population and where road transport is not necessarily the dominant form of transport. We contribute to this research gap by presenting a FE typology for the Pacific Region that captures the diversity of physical spaces, social connections and pathways or mechanisms through which food is commonly acquired. We test this typology and analyse the relative importance of these different FEs in people’s diets in the Solomon Islands. Greater understanding of the relative importance of the different places and ways people acquire food will enable the identification of leverage points to improve the FE to achieve improved health outcomes for the population in future. Specifically, this paper addresses the following research questions:

1. What and where are the different points of food acquisition (FEs) in the Pacific food system and what are the common mechanisms of exchange?
2. What is the relative importance of these FEs in diets for different population groups in Solomon Islands?
3. What is the relationship between reliance on different FEs and dietary quality in Solomon Islands?

## 2. Methods

### 2.1. Conceptual Typology of Food Environments

In order to describe the sources of food acquisition in the Pacific region, we adapt existing conceptual frameworks of FEs [12,14,26] to the Pacific context. The framework by Turner et al. [12] distinguishes between the ‘external’ and ‘personal’ FE whereby the external domain includes characteristics such as food availability, prices and vendor properties, whilst the personal FE is determined by individual level factors that interact with the external FE such as accessibility, affordability and desirability. Whilst they recognise four specific food sources relevant to LMICs as informal markets, own production, wild harvest and transfers, there is no elaboration on the variety of FEs within these categories. Downs et al. [14] build on this by presenting four major categories of FEs relevant to LMICs and HICs, including wild, cultivated, informal and formal retail, and 21 sub-types. However, they do not describe personal or community relationships, nor do they describe food aid, as sources of food. Haynes et al. [26] present food sources relevant in two small case study communities from small island states including one in the Pacific region, again, building on existing typologies, and focusing on the exchange mechanism (purchase, own production, etc.) as a distinguishing feature.

Revisions were informed by the research team’s cumulative field experience in understanding regional and local agri-food systems in the Pacific region representing 22 countries and territories across Micronesia, Melanesia and Polynesia (e.g., [27,28,29,30,31]). The resulting typology of FEs specific to the region elevates the role of community and kinship in the acquisition of food and provides further detail on the exchange mechanisms (e.g., purchase, gifting and so forth) used. Peer review by seven key stakeholders from the region from a diverse array of disciplines including nutrition, trade, fisheries and agriculture was used to ground truth the categorization developed.

### 2.2. Secondary Analysis of Food Acquisition in the Solomon Islands

To further test the adapted typology, and to describe the relative importance of different FEs in people’s diets, we analysed food acquisitions recorded in the Solomon Islands 2012/13 Household Income and Expenditure Survey (HIES) [32]. The survey included a nationally representative sample of 4478 households where diaries were used to record the quantity, value and source of all foods acquired by households over a two-week period and reflects the most recent nationally representative data available on food acquisition for the Solomon Islands. Data collection was staggered over 12 months to account for seasonal changes to consumption patterns. The diary was completed by household members and enumerators visited households every second day for the 14-day period to ensure data was recorded appropriately. The diary format allowed the ‘source’ of each individual food item to be recorded using ‘free text’ by household members. For items which were not acquired through a cash purchase (such as foods harvested from the wild or from home cultivation), economic values were estimated by the respondent based on what the household would have paid in a retail environment. Households were provided with a weighing scale to collect home production volumes in a standard unit of measurement. The sampling procedure for the survey was based on a stratified two stage sampling design. The country was divided into ten strata based on the provinces. From each stratum, primary sampling units (enumeration areas) were selected with a probability proportional to the population size based on the 2009 census. Enumeration areas were selected covering both urban and rural areas within a province. One province (Honiara) had no rural areas and another province (Rennell-Bellona) had no urban areas. From each enumeration area, secondary sampling units (households) were selected. This study obtained ethical approval from the CSIRO Social and Interdisciplinary Science Human Research Ethics Committee (035/21).

#### 2.2.1. Data Preparation

Observations were excluded if the acquisition did not directly benefit the surveyed household (e.g., foods purchased, harvested or collected which were gifted to other households or used for the household’s business); and if the acquisition was of non-food items such as tobacco and narcotics (including kava, which is considered a narcotic under the Classification of Individual Consumption According to Purpose system). This did not exclude any households from the analysis, only those specific transactions by each household. Outliers for quantities of individual food items were identified using Tukey’s inter-quartile range (IQR) method [33] and a multiplier of 1 on log-transformed variables. Outliers (approximately 5% of observations) were replaced with the median quantity of non-outlier data for that food item based on urban or rural status and province.

To account for variation in household composition (number of household members of different ages and gender) we present results as quantity or value of food acquired per adult male equivalent (AME). This allows for a more accurate representation of consumption based on individual energy requirements of household members, compared to per capita estimates [34]. AMEs are calculated by estimating the energy requirements of each household member (based on age and sex) and presenting these as a proportion of the energy requirements of an adult male aged 20–34 years [35]. The AMEs of individual household members are summed to generate a HH level AME and this is used as the denominator in presenting food quantities and expenditure. Results are presented at national level, by urban/rural status, by province and wealth quintile (based on expenditure).

#### 2.2.2. Coding of ‘Source’ Data according to Typology of FEs

**Figure 1:**
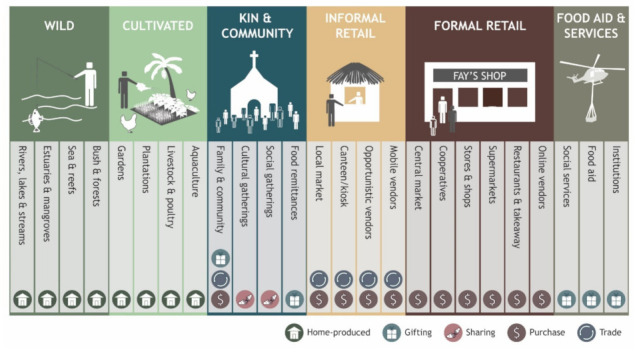
Conceptual typology of food environments in the Pacific Region and primary exchange mechanisms.

In this study we examined the data on ‘source’ recorded in food diaries and categorised each free text listing according to the adapted typology. Of the six main FE types and 25 sub-types presented in the typology (see Table 1 and Figure 1), one main (food aid and services) and six FE sub-types were not able to be deduced from the survey data. To the best of our knowledge, online vendors did not exist in Solomon Islands at the time of the survey and so it is unlikely that this was an important food source (it is, however, included in our typology as this is known to be a growing source of food acquisition in the region broadly and more recently). Similarly, no observations related to aquaculture were identified, though this is also known to be a growing food production system in the region more broadly. Food remittances were not recorded explicitly in the survey, though this is known anecdotally to be an important source of food (and would be captured within ‘gifted’ food). It is likely that if a household received food from geographically distant family or kin during the survey period, the source was recorded as simply a family or friend’s name in the food diary and therefore would be captured under kin and community in this analysis. Social gatherings and cultural gatherings were combined into a single category due to difficulty in separating the free text descriptions of these occasions within the survey data. Very few observations were identified related to livestock and poultry, so these were combined with gardens and subsistence production or plantations and commercial production as relevant. Similarly, mobile vendors were combined with opportunistic vendors due to lack of detail in food source descriptions in order to accurately separate these categories.

JB (primary author) did coding of raw data manually on 85% of observations. Categorisation of the remaining 15% of observations was made by experts from Solomon Islands familiar with the sites and the types of FE. Cross-checking of observations coded by JB was done on 2% of observations (*n* = 5126) by local experts and were found to be highly consistent (87% consistent). Observations for which the source was missing, incomprehensible (such as listings of numbers or symbols) or otherwise not possible to categorise within the typology were coded as ‘undetermined’ and included in analysis so as not to underrepresent total food quantity or expenditure. These include diary entries for pocket money given to children mostly for purchases at school. Given that it is not possible to determine what types of food environments this money was spent within (e.g., school canteens, or opportunistic vendors located near schools), these entries were considered undetermined. Due to data limitations, the exchange mechanisms are presented according to three collapsed categories (rather than the five presented in the typology); (1) purchase or trade, (2) gifting or sharing, and (3) home-produced.

#### 2.2.3. Statistical Analysis

**Figure 2:**
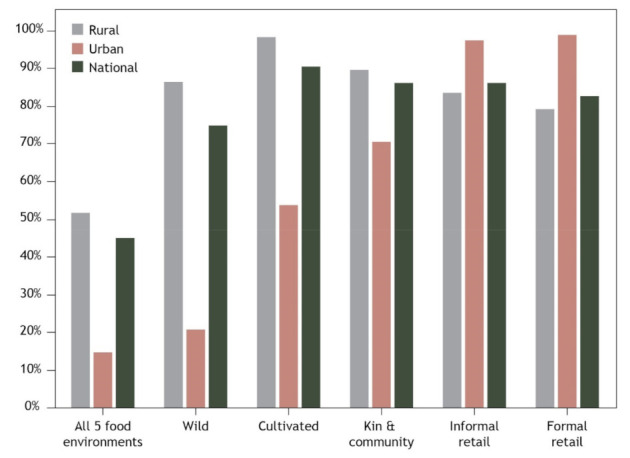
Proportion of households accessing different food environments in Solomon Islands, nationally and in urban and rural areas.

Data were analysed using Stata version 13. The ‘svyset’ command in combination with probability weights were used to account for the complex survey data structure. The proportion of households accessing various FEs (Figure 2, Supplementary Material Table S1) was estimated using the svy: proportion command. Differences between urban and rural households were tested for statistical significance using lincom command (*t*-test) with significance level of *p* < 0.05. Differences between provinces was tested using logistic regression followed by the pwcompare (pairwise comparisons) command and the Bonferonni adjustment for multiple comparisons with significance level of *p* < 0.05. To characterise the relationship between reliance on different FEs (for acquisition of food) and diet quality (using food acquisition as proxies for diet quality) we conducted multivariate regression analyses. The primary outcome variables were 1) quantity of fruits and vegetables acquired in grams/AME/day and 2) quantity of ultra-processed foods (UPFs) acquired in grams/AME/day. UPFs were identified according to the NOVA food classification system [36]. The predictor variables were the reliance on each FE reflected by a binary variable (sourced any quantity of food from that FE during the survey period, did not source any food from that FE during the survey period). Multivariate models controlled for the following potential confounding variables: urban/rural location of households, gender of head of household, education level of household head (three categories; nil or incomplete primary education, primary, secondary or higher education), wealth group (expenditure quintiles), age of household head and household size (number of members). Both outcome variables were positively skewed (did not meet the assumption of a Normal distribution required for linear regression) so a sensitivity analysis was carried out on log transformed outcome variables (which produced a Normal distribution). For the most part this did not change interpretation of results. Results are presented for untransformed outcome variables. In cases where interpretation of results would differ based on log-transformed model, results are marked with an asterisk and explained as a footnote to Table 2.

## 3. Results

### 3.1. Food Environment Typology

The Pacific typology of FEs includes six main FE types; wild, cultivated, kin and community, informal retail, formal retail and food aid and services; and 25 sub-types (Figure 1, Table 1). Wild FE sub-types considered here include bush and forests, sea and reefs, estuaries and mangroves, and rivers, lakes and streams. The cultivated FE in the Pacific region includes gardens, plantations, livestock and poultry and aquaculture. The latter three are only considered a ‘FE’ when those involved in production use some of the produce for direct household consumption (excluding those who source this food via an alternative pathway such as formal retail). It is recognised that very seldom do landscapes remain completely free from human influence and that wild FEs are part of a continuum alongside cultivated FEs in terms of management intensity (see for example [37]).

As noted by [14], the retail FE (also known as the built or market environment) includes both formal and informal settings differentiated by the presence (or absence) of formal governance structures surrounding operations. Formal retail FE subtypes considered here include online vendors, restaurants and takeaway, supermarkets, stores and shops, cooperatives and central markets. Informal retail FE sub-types include local markets, canteens, opportunistic vendors and mobile vendors. It should be noted that the distinction between formal and informal retail environments in terms of regulation is often not clear-cut and operates along a continuum rather than a dichotomy. For example, local markets in some parts of the Pacific region are likely to be guided by some form of governance structure though the degree to which this functions is likely to vary widely across and within countries.

The inclusion of kin and community, and food aid and services FEs are important additions in the Pacific context. We define the kin and community FE as the network of social relationships through which people acquire food. We consider four sub-types of kin and community (as defined in Table 1), family and community (such as exchanging food with relations or neighbouring households), cultural gatherings or ceremonies (such as religious ceremonies), social gatherings (such as hosting guests in a household) and food remittances. Food aid and services is defined as the provision of food from government or non-government organisations in response to acute or chronic food insecurity or as part of institutional food provision, and includes three sub-types; food aid (such as disaster relief in acute food shortages); social services (such as food assistance provided by governments to vulnerable groups facing chronic food insecurity); and institutions (such as the provision of food in hospitals, workplaces, schools, prisons and other institutions).

It should be noted that clear distinctions between the FE types and sub-types presented here cannot always be clearly made and in many cases the typology should be interpreted more as a continuum rather than isolated categories. For example, fruit trees grown in areas within or nearby villages might be minimally tended by community members but do not fall neatly within the wild or cultivated FEs.

Alongside this typology we present the primary exchange mechanisms through which food is most likely to be acquired in the various FEs (see Figure 1 and Table 1). We consider five primary exchange mechanisms relevant to the Pacific region defined as follows:

(1) Purchase: to acquire through a monetary transaction such as cash or electronic funds transfer.
(2) Home produced: to acquire food produced by household members using their own capital and unpaid labour.
(3) Gifting: acquired through social norms or customs without any exchange of money, goods or services. This is a one-way exchange where one party gifts the food, and the other party receives it.
(4) Trading: acquired through a non-monetary exchange of goods or services such as the exchange of food items for labour, or other food items.
(5) Sharing: similar to gifting, but reflects occasions where the food is consumed immediately, and the ‘giver’ participates in consumption. One or several giving groups contribute to the ‘pool’ of food which is then shared communally. Sharing is typically associated with social and cultural functions including receiving guests in a household.

### 3.2. HIES Analysis

#### 3.2.1. Diversity of Food Environments

Of the 16 FE sub-types explored in this survey, on average, households accessed 6.4 different FE sub-types, with some regional variability (Supplementary Material Table S1). Rural households accessed a slightly but significantly greater diversity of FEs compared to urban households (mean 6.5 and 5.7, respectively, *p* < 0.05). Households in Rennell-Bellona accessed a significantly lower diversity of FE sub-types compared to all other provinces (mean 5.3, *p* < 0.05). Wealthier households accessed a slightly higher number of FEs on average, compared to the lowest wealth group.

As would be expected, nearly all urban households accessed the formal (99%) and informal (98%) retail FE compared to 79% and 84% of rural households respectively (*p* < 0.05, Figure 2, Supplementary Material Table S1). In contrast, nearly all rural households accessed the cultivated (98%) and wild (87%) FE compared to 54% and 21% of urban households respectively (*p* < 0.05). Most households rely to some extent on kin and community as a source of food, accessed by 71% of urban and 89% of rural households.

Access to different FE types varied widely between provinces, with the extremes typically reflected by differences between Rennell-Bellona (an exclusively rural area) and Honiara (an exclusively urban area, Supplementary Material Table S1). Rennell-Bellona had the lowest proportion of households accessing formal and informal retail FEs but the highest proportion of households accessing the cultivated and kin and community FEs. In contrast, Honiara had the highest proportion of households accessing the formal and informal retail FEs, and the lowest accessing the cultivated, wild and kin and community FEs.

The proportion of households accessing food via all five FE types varies widely; 45% nationally compared to 15% of urban households and 52% of rural households (Figure 2, Supplementary Material Table S1). The lowest access to all five FEs was within Honiara (3% of households) and Rennell-Bellonna (16%) indicating that access to various FE types is linked to both urban and rural contexts. The greatest diversity of access was observed in Choiseul province where 63% of households accessed all five FEs. No clear trend was observed amongst wealth groups, with the highest proportion of households accessing all five FEs from the middle wealth group.

Access to different FE types also differs by wealth group (Supplementary Material Table S1). As wealth increases, the proportion of households accessing formal and informal retail FEs also increases. In contrast, as wealth increases the proportion of households accessing the cultivated and wild FE decreases significantly (*p* < 0.05). A slighter higher proportion of households in lower wealth groups accessed kin and community FEs though differences were not significant across wealth groups.

#### 3.2.2. Food Acquisition by Food Environment Type

**Figure 3:**
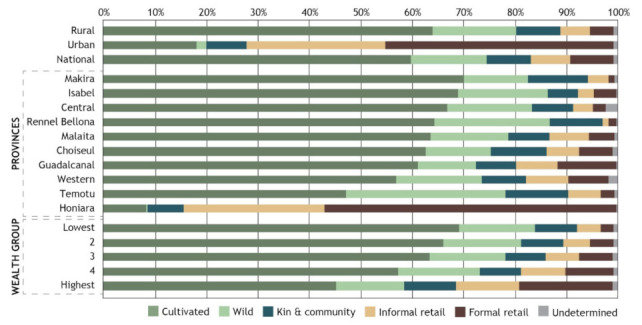
Proportion of total quantity of food acquired from different food environments Nationally, by geographic location and wealth groups.

The cultivated FE is by far the most important FE, accounting for 60% of the quantity of food acquired nationally (Figure 3, Supplementary Material Table S2). This is followed by the wild FE (15%), kin and community (9%), and formal and informal retail FEs (8% each). When examining FE sub-types, gardens account for the largest quantity of food acquisition, followed by family and community, and sea and reefs (8% each, Supplementary Material Table S2). Perhaps surprisingly, central, and local markets account for only 3% and 4% of the total quantity of food acquired, respectively.

As expected, there was wide variation in the relative importance of FE types and sub-types according to urban vs. rural areas, as well as across provinces. The formal and informal retail environments dominate food acquisition in terms of quantity in urban areas whilst the wild and cultivated FEs play a much more significant role in rural areas and most of the provinces (excluding Honiara which is an exclusively urban area). The importance of kin and community is much more consistent (less variable) across the provinces and urban vs. rural areas compared to other FE types.

As expected, there is also wide variation in dependence on different FEs according to wealth groups. Least wealthy households rely much more heavily on the cultivated FE compared to wealthy households (70% and 45% of the quantity of food acquired respectively). The opposite trend is seen in reliance on the formal retail FE; households from the lowest wealth group acquired 3% of their total food (by quantity) from formal retail compared to 18% of total food acquired by wealthy households. Interestingly, reliance on wild and kin and community FEs was relatively consistent across wealth groups.

When food acquisition by FE types is examined according to the economic value of food (Supplementary Material Table S3, rather than quantity as above), some slightly different patterns emerge. The cultivated FE still accounts for the vast majority of food acquired nationally, albeit at a lower level (33% of food acquisition) followed by formal retail (27%), informal retail (14%), and kin and community and wild FEs (12% each). These differences could reflect differences in the types of food items accessed through the different FEs (e.g., that foods commonly sourced from the cultivated FEs are cheaper per unit compared to food items commonly sourced from retail FEs). Alternatively, this could also indicate underreporting of economic value of foods from cultivated FEs at the point of data collection.

#### 3.2.3. Mechanisms of Exchange

As expected, home production is the dominant exchange mechanism for foods acquired from the wild and cultivated FEs accounting for 98% and 99% of transactions of food acquired, respectively (data not shown). As expected also, purchase or trade is the predominate exchange mechanism for food acquired from both the formal and informal retail environments accounting for 100% and 99% of transactions, respectively. The exchange mechanism for foods acquired through the kin and community FE is mixed with gifting or sharing accounting for 60% of transactions and the remaining 40% acquired through purchase or trade mechanisms. This contributes further evidence that kin and community is a key structural component of the FE in Solomon Islands.

#### 3.2.4. Food Environments and Diet Quality

**Figure 4:**
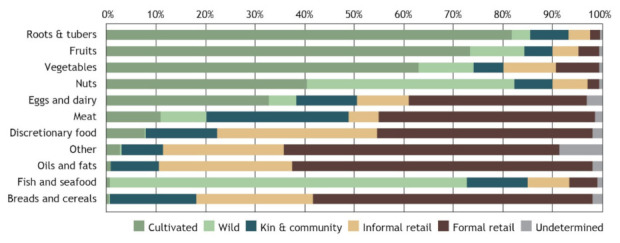
Proportion of total quantity of food groups acquired from different food environments in Solomon Islands nationally.

The cultivated FE provides the majority of roots and tubers (82%), fruits (73%) and vegetables (63%), as well as a considerable proportion of nuts (41%) and eggs (33%) acquired nationally (Figure 4, Supplementary Material Table S4). In contrast, formal retail provides the majority of oils and fats (60%), breads and cereals (56%), meat (43%), and discretionary food (43%). The wild FE provides the majority of fish and seafood (72%), and nuts (42%) acquired nationally. Kin and community play a more moderate role across several food groups as a source of meat (29%), breads and cereals (18%), discretionary food (14%) and fish and seafood (12%). Similar trends can be seen when examining the proportion of food groups acquired from different FEs in terms of economic value of food (rather than quantity, Supplementary Material Table S5), though with some noticeable deviations. For example, formal and informal retail together account for 14% of the quantity of fish and seafood acquired, but 35% of the value of seafood acquired.

Reliance on different FEs are significant predictors of diet quality (two separate measures as proxies) after controlling for potential confounders (Table 2, Supplementary Material Table S6). Reliance on cultivated, wild and kin and community FEs are significant positive predictors of fruit and vegetable acquisition. The effect size was largest for wild FEs; households that relied on wild FEs acquired 239 g more fruit and vegetables/AME/day compared to households that did not rely on wild FEs (*p* < 0.05). In contrast, reliance on formal retail is a significant negative predictor of fruit and vegetable acquisition (β = −179, *p* < 0.05). No significant relationship was found between reliance on informal retail and fruit and vegetable acquisition. When examining UPF acquisition, reliance on formal and informal retail were both significant and positive predictors, whilst reliance on the wild FE was a significant negative predictor. No significant relationship was found between reliance on cultivated or kin and community FEs and UPF acquisition.

## 4. Discussion and Conclusions

We present a novel typology of FEs for the Pacific region that captures the variety of places and pathways through which people acquire food. This includes six main FE types; wild, cultivated, kin and community, informal retail, formal retail and food aid and services; and 25 sub-types. We make several important contributions beyond existing FE frameworks including: (1) the inclusion of kin and community FE; (2) the inclusion of food aid and services FE; and (3) articulation of the primary exchange mechanisms through which food is typically acquired from these FEs in the Pacific, such as purchase, home produced, trade, gifting or sharing. These additions are likely to be applicable to FEs beyond the Pacific region, particularly in LMICs.

Home production is the predominant exchange mechanism within wild and cultivated FEs, whilst purchase or trade is the predominant exchange mechanism within formal and informal retail FEs. Food aid and services FEs are based solely on gifting (by definition) as the transaction mechanism and are likely to be a small but notable feature of FEs in the Pacific Region given the role they play in prevention of malnutrition, particularly in times of acute food shortages that can occur following events such as natural disasters. Kin and community FEs feature several exchange mechanisms including purchase, trade, gifting and sharing. They reflect the historical importance of trade between local tribal or kinship groups with access to different food resources such as the trade of root vegetables and fish between hill and sea people [38]. The kin and community FE also captures the importance of social and cultural gatherings as a source of food including gifting or sharing foods within extended family groups on a daily basis, and the more significant cultural and ceremonial events that feature regularly in the lives of Pacific Islanders. Kin and community as a source of food is not unique to the Pacific region. Similar patterns of trade of diverse foods across agroecological zones in close proximity are found elsewhere including South America where this phenomenon is described as a ‘vertical archipelago’ [39]. Friends and neighbours as an important food source has also been recognised in many contexts around the world (for example Indonesia [40] and Mexico [41]), indicating the relevance of this FE typology beyond the Pacific region.

We then applied this typology to analysis of a large nationally representative survey of food acquisition in Solomon Islands to quantify, for the first time, the reliance of different population groups on different FEs and the role they play in contributing to diet quality. This is a significant contribution to the literature as there are few studies that examine the relationship between FE types and diet quality in the Pacific region (e.g., [26,42]) and no studies to our knowledge that examine this relationship across all the relevant FEs presented here. The cultivated FE is by far the most important source of food in Solomon Islands providing 60% of the quantity and 33% of the value of food acquired nationally. We quantified for the first time, the contribution of wild food to food acquisition nationally, accounting for 15% of the quantity of food and 12% of the value of food acquired, the majority of which is sourced from marine environments. Kin and community are also a largely unrecognised but important source of food accounting for 9% of the quantity and 12% of the value of food acquisition nationally. Perhaps surprisingly, central and local markets make a smaller contribution to food acquisition, together accounting for 7% of food acquired nationally (by both quantity and value).

Reliance on different FEs are significant predictors of diet quality. Reliance on formal retail FEs was associated with lower diet quality on both measures, lower fruit and vegetable acquisition and higher UPF acquisition. In contrast, reliance on cultivated, wild and kin and community FEs are significant positive predictors of fruit and vegetable acquisition. This was supported by the descriptive analysis which shows that cultivated and wild FEs provide the overwhelming majority of roots and tubers, fruits, nuts, vegetables, and fish and seafood, in contrast to formal and informal retail FEs which provide the overwhelming majority of oils and fats, discretionary foods and breads and cereals.

The application of this FE typology to food acquisition data provides crucial information on how to target initiatives that aim to improve the quality of diets in the Pacific region to address malnutrition and the associated rising prevalence of NCDs. For example, in the Solomon Islands initiatives that support the cultivation of fruits and vegetables will be crucial to increasing consumption of these foods for most of the population. Similarly, initiatives that support fisheries management approaches and the natural resources on which fisheries depend will be crucial for the sustainable provision of fish and seafood from wild sources. Such initiatives will be crucial in supporting the nutritional benefits of aquatic foods including the provision of high-quality animal protein, bioavailable micronutrients and essential fatty acids that reduce the risk of NCDs such as cardiovascular disease [43,44]. Actions to reduce consumption of discretionary, and particularly UPFs must target shops, stores and canteens. Actions must also be targeted separately to urban and rural areas where the reliance on different FEs varies widely. Future studies should seek to further characterise these different FE types and subtypes in terms of availability, affordability, promotion, quality, convenience and sustainability of foods. Such assessments must be underpinned by the development of valid and reliable measurement tools to capture these dimensions across the variety of FE types, across contexts and over time. Understanding how these characteristics of FEs shape dietary patterns will provide crucial information for the design of interventions within the FE to improve health and sustainability of diets in the region.

A limitation of this analysis is that in some cases the level of detail regarding the source of food recorded in survey data was not always able to be clearly delineated within the FE typology. For example, it is relatively common for households to operate small canteens from within or attached to the house allowing neighbours to purchase or trade food. In the survey this source of food might be recorded simply by a person’s name (considered kin and community within this typology) or described as a canteen (informal retail). This means that the reliance on different FEs may be slightly over or underrepresented. Furthermore, although we use the most recent HIES data available (2012/13), it is likely that diets have continued to evolve during this time. Future analyses should build in a typology of FEs to the data collection stages and be conducted with a greater frequency to improve the accuracy of these assessments. Another limitation is that we use household level food acquisition (specifically, fruit and vegetable acquisition, and UPFs acquisition) as imperfect proxies for diet quality. Future studies should investigate the relationship between food environments and diet quality based on actual consumption surveys, preferably at the individual level.

Lack of high-quality large-scale individual-level dietary surveys to understand dietary patterns remains a significant challenge for the appropriate design of interventions to improve diets and nutrition. An under-utilised data source that partly addresses these gaps are HIES surveys [45,46]. These are periodically carried out (often every 5–10 years) at national scales and can collect relatively detailed data on food acquisition. It is commonplace for HIES surveys to collect data on the source of food acquisition according to purchases, home-produced, or gifted transactions. Expansion of these options to reflect a FE typology such as the one presented here would incur a very small burden for data collection but create a wealth of data to understand where people source foods and therefore better inform interventions to improve diets.

The design of policies and interventions to reduce the burden of malnutrition and NCDs in the Pacific region will require deeper understanding of the characteristics of the FE as a driver of diet quality, including how this varies within and throughout the region for different population groups. The typology presented here provides a framework for examining these relationships in the Pacific and is likely applicable to LMICs more broadly. Our analysis reaffirms the importance of subsistence agriculture and the cultural basis of food acquisition through community and kinship networks (known as wantok in some parts of the Pacific region [47]). At the national scale, interventions in the Solomon Islands food system to support and sustain wild and cultivated FEs as key sources of roots and tubers, fruits, nuts, vegetables, and fish and seafood is likely to be more impactful than over-emphasis on formal retail FEs. The national policy response to the COVID 19 pandemic reinforced the central role of cultivated and wild FEs in Solomon Islands. Under the State of Emergency declared in March 2020, urban dwellers were encouraged to return to home villages and informal retail FEs were closed [48]. These changes and the internal migration that followed prompted an increase in gardening and community cooperation [48]. Although the survey data used in this analysis are nearly a decade old, it is clear that cultivated and wild FEs, and the production systems and social structures that sustain them, remain an important source of resilience in Solomon Islands.

## Figures and Tables

**Table 1:** Definitions of food environment sub-types in the Pacific.

Food Environment Sub-Type	Description	Primary Exchange Mechanism	Relationship between Acquisition and Consumption
**Wild**			
Rivers, lakes and streams	Food harvested from freshwater sources that have been produced without (or with minimal) human management or input.	Home produced	Food usually acquired in advance and then taken to the household for preparation and consumption
Estuaries and mangroves	Food harvested from estuary sources that have been produced without (or with minimal) human management or input.	Home produced	
Sea and reefs	Food harvested from marine sources that have been produced without (or with minimal) human management or input.	Home produced	
Bush and forests	Food harvested from terrestrial sources that have been produced without (or with minimal) human management or input.	Home produced	
**Cultivated**			
Gardens	Foods grown in a household or family plot of land such as gardens and located near or far from the household.	Home produced	Food usually acquired in advance and then taken to the household for preparation and consumption
Plantations	Foods grown in cultivated plots primarily for commercial sale (if used as a source for own consumption) and located near or far from the household.	Home produced	
Livestock and poultry	Livestock and poultry raised either on household plots or dedicated land for commercial sale (if used as a source for own consumption).	Home produced	
Aquaculture	Aquatic foods cultivated in purpose-built structures (such as ponds) or modifications to natural water bodies (such as rock pools or cages).	Home produced	
**Kin and community**			
Family and community	Members of the local community including family members, where a person has some form of personal connection that enables the food transaction, e.g., community members visit neighbouring households as required to purchase or trade food items.	Purchase, trade, gifting	Food usually acquired in advance and then taken to the household for preparation and consumption
Food remittances	Food sent long distances (between provinces or internationally) usually between family members.	Gifting	
Cultural gatherings	Community members come together for cultural, religious or ceremonial reasons and share food.	Sharing	Food usually consumed at point of acquisition
Social gatherings	Visiting or receiving guests from another household (for social reasons) and sharing food.	Sharing	
**Informal retail**			
Local market	Markets that occur either in provincial capitals (but excluding the primary markets) or in other regions. These markets include multiple vendors in an open-air communal area either with no roof, or individually managed temporary umbrellas or thatched roofs.	Purchase, trade	Mixed
Canteen or kiosk	Semi-permanent structure such as an open-fronted kiosk or hut where customers stand outside the hut and request items to purchase from the vendor. Sometimes these are attached to, or are part, of houses.	Purchase, trade	
Opportunistic vendors	Temporary vendors that set up with no or minimal equipment such as a tarp or small table and sell items opportunistically at certain times of the day or week, e.g., boat landing sites, walking trails, or roadsides.	Purchase, trade	
Mobile vendors	Temporary vendors that use minimal equipment (such as baskets) to sell food whilst roaming from place-to-place.	Purchase, trade	
**Formal retail**			
Online vendors	Food purchased online usually with a smart phone or app and delivered to consumers.	Purchase	Food usually consumed at point of acquisition
Restaurants and takeaway	A permanent structure where pre-prepared meals, snacks and beverages are sold for immediate consumption either on-site or for takeaway.	Purchase	
Supermarkets	A large permanent structure, often a ‘chain’ store, selling a large variety of fresh and processed food products with items displayed in aisles, often including refrigerated sections.	Purchase	Food usually acquired in advance and then taken to the household for preparation and consumption
Stores and shops	A permanent structure (smaller than a supermarket) where customers can enter the store and choose items from shelves in a self-serve manner. A smaller selection of mostly packaged foods compared to supermarkets.	Purchase	
Cooperatives	A store which is operated and run by a community of people or members where benefits are shared.	Purchase	
Central market	The primary market in urban centres or provincial capitals. These markets include multiple vendors in a semi-permanent open-air communal area usually under a single roof (or immediately adjacent to).	Purchase	
**Food aid and services**			
Social services	Food provided by governments on a regular and consistent basis to vulnerable population groups experiencing poverty and or food insecurity.	Gifting	Food usually acquired in advance and then taken to the household for preparation and consumption
Food aid	Food relief provided by governments or NGOs in response to short term food system shocks such as natural disasters.	Gifting	
Institutions	Food provided within public or private institutions such as schools, workplaces, hospitals, aged care facilities, child-care facilities, prisons, and others.	Gifting	Food usually consumed at point of acquisition

**Table 2:** Effect of reliance on different FEs (independent variables) on two measures of diet quality (fruit and vegetable acquisition, and ultra-processed food acquisition).

	Outcome Variable: Fruit and Vegetable Acquisition (g/AME/day)						
Independent Variables	Direction and Nature of Relationship	β Coefficient	SE	t	*p* Value	CI Lower Bound	CI Upper Bound
Reliance on formal retail FE	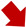	−178.84	50.10	−3.57	0.001	−278.14	−79.55
Reliance on informal retail FE	-	−37.65	52.32	−0.72	0.473	−141.34	66.04
Reliance on cultivated FE	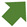	118.70	36.17	3.28	0.001	47.02	190.38
Reliance on wild FE	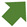	239.08	40.22	5.94	<0.001	159.36	318.81
Reliance on kin and community FE	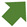	192.01	38.58	4.98	<0.001	115.56	268.47
	**Outcome Variable: UPFs Acquisition (g/AME/day)**						
Reliance on formal retail FE	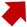	8.04	1.41	5.69	<0.001	5.24	10.84
Reliance on informal retail FE	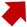	5.47	1.45	3.78	<0.001	2.61	8.34
Reliance on cultivated FE	-	−2.68	2.63	−1.02	0.310	−7.90	2.54
Reliance on wild FE	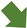	−3.59	1.89	−1.90	0.060 *	−7.33	0.16
Reliance on kin and community FE	-	0.40	2.09	0.19	0.847	−3.74	4.55

Green arrow indicates statistically significant relationship (*p* < 0.05) which is positive for diet quality. Red arrow indicates statistically significant relationship (*p* < 0.05) which is negative for diet quality. Full regression model available in Supplementary Material Table S6. FE, food environment. SE, standard error. CI, confidence interval. AME, adult male equivalent. UPF, ultra-processed food. * *p*-value for log-transformed outcome variable was *p* = 0.008.

## Data Availability

Solomon Islands 2012/13 Household Income and Expenditure Survey data is available on request from Solomon Islands National Statistics Office.

## Supplementary Materials

The following are available online at https://www.mdpi.com/article/10.3390/foods10112592/s1, Table S1: Diversity of food environments utilised Nationally, by geographic location and wealth groups; Table S2: Proportion of total quantity of food acquired from different food environments Nationally, by geographic location and wealth groups; Table S3: Proportion of total value of food acquired from different food environments Nationally, by geographic location and wealth groups; Table S4: Proportion of food groups (quantity) acquired from different food environments in Solomon Islands nationally; Table S5: Proportion of food groups (value) acquired from different food environments in Solomon Islands nationally; Table S6: Effect of reliance on different FEs (independent variables) on two measures of diet quality (fruit and vegetable acquisition, and ultra-processed food acquisition), full model including potential confounders.

## References

[1] Development Initiatives (2020). Global Nutrition Report: Action on Equity to End Malnutrition.

[2] Willett W., Rockström J., Loken B., Springmann M., Lang T., Vermeulen S., Garnett T., Tilman D., DeClerck F., Wood A. (2019). Food in the Anthropocene: The EAT-Lancet Commission on healthy diets from sustainable food systems. Lancet.

[3] Swinburn B.A., Kraak V.I., Allender S., Atkins V.J., Baker P.I., Bogard J.R., Brinsden H., Calvillo A., De Schutter O., Devarajan R. (2019). The Global Syndemic of Obesity, Undernutrition, and Climate Change: The Lancet Commission report. Lancet.

[4] Taft K. (2020). Leading Advocates for Transforming Global Food Systems Named Ahead of Milestone UN Summit.

[5] FAO, WHO (2014). Framework for Action: Outcome Document. Proceedings of the Second International Conference on Nutrition.

[6] HLPE (2017). Nutrition and Food Systems. A Report by the High Level Panel of Experts on Food Security and Nutrition of the Committee on World Food Security.

[7] FAO, IFAD, UNICEF, WFP, WHO (2020). The State of Food Security and Nutrition in the World 2020. Transforming food Systems for Affordable Healthy Diets.

[8] FAO, WHO (2016). United Nations Decade of Action on Nutrition 2016–2025.

[9] United Nations (2015). Transforming Our World: The 2030 Agenda for Global Action.

[10] Kennedy G., Rota Nodari G., Trijsburg L., Talsma E., Haan S.d., Evans B., Hernandez R., Achterbosch T. (2020). Compendium of Indicators for Food System Assessment.

[11] Turner C., Kalamatianou S., Drewnowski A., Kulkarni B., Kinra S., Kadiyala S. (2020). Food Environment Research in Low- and Middle-Income Countries: A Systematic Scoping Review. Adv. Nutr..

[12] Turner C., Aggarwal A., Walls H., Herforth A., Drewnowski A., Coates J., Kalamatianou S., Kadiyala S. (2018). Concepts and critical perspectives for food environment research: A global framework with implications for action in low- and middle-income countries. Glob. Food Secur..

[13] Lytle L.A., Sokol R.L. (2017). Measures of the food environment: A systematic review of the field, 2007–2015. Health Place.

[14] Downs S.M., Ahmed S., Fanzo J., Herforth A. (2020). Food Environment Typology: Advancing an Expanded Definition, Framework, and Methodological Approach for Improved Characterization of Wild, Cultivated, and Built Food Environments toward Sustainable Diets. Foods.

[15] Sallis J.O.N., Fisher E., Glanz R.B.K., Viswanath K. (2008). Ecological Models of Health Behavior. Health Behavior and Health Education: Theory, Research, and Practice.

[16] Swinburn B.A., Sacks G., Hall K.D., McPherson K., Finegood D.T., Moodie M.L., Gortmaker S.L. (2015). The global obesity pandemic: Shaped by global drivers and local environments. Lancet.

[17] Glanz K., Sallis J.F., Saelens B.E., Frank L.D. (2007). Nutrition Environment Measures Survey in stores (NEMS-S): Development and evaluation. Am. J. Prev. Med..

[18] Herforth A., Ahmed S. (2015). The food environment, its effects on dietary consumption, and potential for measurement within agriculture-nutrition interventions. Food Secur.

[19] Development Initiatives (2020). Global Nutrition Report: Action on Equity to end Malnutrition. Regional Overview: Oceania.

[20] IDF (2019). IDF Diabetes Atlas.

[21] Santos J.A., McKenzie B., Trieu K., Farnbach S., Johnson C., Schultz J., Thow A.M., Snowdon W., Bell C., Webster J. (2019). Contribution of fat, sugar and salt to diets in the Pacific Islands: A systematic review. Public Health Nutr..

[22] Thow A.M., Heywood P., Schultz J., Quested C., Jan S., Colagiuri S. (2011). Trade and the Nutrition Transition: Strengthening Policy for Health in the Pacific. Ecol. Food Nutr..

[23] Dancause K.N., Dehuff C., Soloway L.E., Vilar M., Chan C., Wilson M., Tarivonda L., Regenvanu R., Kaneko A., Garruto R.M. (2011). Behavioral changes associated with economic development in the South Pacific: Health transition in Vanuatu. Am. J. Hum. Biol..

[24] Sievert K., Lawrence M., Naika A., Baker P. (2019). Processed Foods and Nutrition Transition in the Pacific: Regional Trends, Patterns and Food System Drivers. Nutrients.

[25] Lee A., Mhurchu C.N., Sacks G., Swinburn B., Snowdon W., Vandevijvere S., Hawkes C., L’Abbé M., Rayner M., Sanders D. (2013). Monitoring the price and affordability of foods and diets globally. Obes. Rev..

[26] Haynes E., Bhagtani D., Iese V., Brown C.R., Fesaitu J., Hambleton I., Badrie N., Kroll F., Guell C., Brugulat-Panes A. (2020). Food Sources and Dietary Quality in Small Island Developing States: Development of Methods and Policy Relevant Novel Survey Data from the Pacific and Caribbean. Nutrients.

[27] Albert J., Bogard J., Siota F., McCarter J., Diatalau S., Maelaua J., Brewer T., Andrew N. (2020). Malnutrition in rural Solomon Islands: An analysis of the problem and its drivers. Matern. Child Nutr..

[28] Farrell P., Thow A.M., Wate J.T., Nonga N., Vatucawaqa P., Brewer T., Sharp M.K., Farmery A., Trevena H., Reeve E. (2020). COVID-19 and Pacific food system resilience: Opportunities to build a robust response. Food Secur..

[29] Andrew N.L., Bright P., de la Rua L., Teoh S.J., Vickers M. (2019). Coastal proximity of populations in 22 Pacific Island Countries and Territories. PLoS ONE.

[30] Farmery A.K., Scott J.M., Brewer T.D., Eriksson H., Steenbergen D.J., Albert J., Raubani J., Tutuo J., Sharp M.K., Andrew N.L. (2020). Aquatic Foods and Nutrition in the Pacific. Nutrients.

[31] Farrell P., Thow A.M., Rimon M., Roosen A., Vizintin P., Negin J. (2021). An Analysis of Healthy Food Access amongst Women in Peri-urban Honiara. Hawaii J. Health Soc. Welf..

[32] Solomon Island National Statistics Office Household Income and Expenditure Survey 2012–2013. https://microdata.pacificdata.org/index.php/catalog/731/study-description.

[33] Tukey J.W. (1977). Exploratory Data Analysis.

[34] Weisell R., Dop M.C. (2012). The adult male equivalent concept and its application to Household Consumption and Expenditures Surveys (HCES). Food Nutr. Bull..

[35] FAO, WHO, UNU (2004). Human Energy Requirements: Report of a Joint FAO/WHO/UNU Expert Consultation.

[36] Monteiro C.A., Cannon G., Levy R.B., Moubarac J.-C., Louzada M.L.C., Rauber F., Khandpur N., Cediel G., Neri D., Martinez-Steele E. (2019). Ultra-processed foods: What they are and how to identify them. Public Health Nutr..

[37] Powell B., Thilsted S.H., Ickowitz A., Termote C., Sunderland T., Herforth A. (2015). Improving diets with wild and cultivated biodiversity from across the landscape. Food Secur..

[38] Ross H.M. (1978). Baegu markets, areal integration and economic efficiency in Malaita, Solomon Islands. Ethnology.

[39] Keleman Saxena A., Cadima Fuentes X., Gonzales Herbas R., Humphries D.L. (2016). Indigenous Food Systems and Climate Change: Impacts of Climatic Shifts on the Production and Processing of Native and Traditional Crops in the Bolivian Andes. Front. Public Health.

[40] Colozza D., Avendano M. (2019). Urbanisation, dietary change and traditional food practices in Indonesia: A longitudinal analysis. Soc. Sci. Med..

[41] Sharkey J.R., Dean W.R., Johnson C.M. (2012). Use of vendedores (mobile food vendors), pulgas (flea markets), and vecinos o amigos (neighbors or friends) as alternative sources of food for purchase among Mexican-origin households in Texas border colonias. J. Acad. Nutr. Diet..

[42] Vogliano C., Raneri J.E., Maelaua J., Coad J., Wham C., Burlingame B. (2021). Assessing Diet Quality of Indigenous Food Systems in Three Geographically Distinct Solomon Islands Sites (Melanesia, Pacific Islands). Nutrients.

[43] Bogard J.R., Farmery A.K., Little D.C., Fulton E.A., Cook M. (2019). Will fish be part of future healthy and sustainable diets?. Lancet Planet. Health.

[44] Golden C.D., Koehn J.Z., Shepon A., Passarelli S., Free C.M., Viana D.F., Matthey H., Eurich J.G., Gephart J.A., Fluet-Chouinard E. (2021). Aquatic foods to nourish nations. Nat. Cell Biol..

[45] Fiedler J.L., Carletto C., Dupriez O. (2012). Still waiting for Godot? Improving Household Consumption and Expenditures Surveys (HCES) to enable more evidence-based nutrition policies. Food Nutr. Bull..

[46] Fiedler J.L., Lividini K., Bermudez O.I., Smitz M.F. (2012). Household Consumption and Expenditures Surveys (HCES): A primer for food and nutrition analysts in low- and middle-income countries. Food Nutr. Bull..

[47] Schram R. (2015). Notes on the Sociology of Wantoks in Papua New Guinea. Anthropol. Forum.

[48] Eriksson H., Ride A., Boso D., Sukulu M., Batalofo M., Siota F., Gomes C. (2020). Changes and Adaptations in Village Food Systems in Solomon Islands: A Rapid Appraisal during the Early Stages of the COVID-19 Pandemic.

