# Supplementary material for: A Typology of Food Environments in the Pacific Region and Their Relationship to Diet Quality in Solomon Islands
Source: Foods. 2021 Oct 27;10(11):2592. doi: 10.3390/foods10112592 (PMC8620377; doi:10.3390/foods10112592)
Supplement: Supplementary file 1 [file foods-10-02592-s001.zip › foods-1365459-supplementary.pdf]

Supplementary Tables and Figures

Supplementary Table S1: Diversity of food environments utilised Nationally, by geographic location and wealth groups.

	% HHs that are urban	% HHs that are rural	Mean number of FE sub-types accessed		% HHs accessing all 5 FEs		% HHs accessing cultivated FE		% HHs accessing wild FE		% HHs accessing kin and community FE		% HHs accessing informal retail FE		% HHs accessing formal retail FE	
			Mean	SE	%	SE	%	SE	%	SE	%	SE	%	SE	%	SE
National	17.6	82.4	6.4	0.06	45.2	1.9	90.6	1.1	74.9	1.8	86.2	1.2	86.0	1.4	82.6	1.6
Urban	100.0	0.0	5.7	0.08	14.7	2.0	53.9	2.8	20.8	2.4	70.6	2.0	97.5	0.6	98.9	0.6
Rural	0.0	100.0	6.5	0.07	51.7	2.1	98.4	0.4	86.5	1.8	89.5	1.4	83.6	1.7	79.1	1.9
**Provinces**																
Choiseul	2.7	97.3	6.6	0.07 [b]	62.6	4.9 [d]	95.8	0.8 [a,b]	85.3	2.0 [a,b]	92.1	1.4 [c,d]	85.1	2.2 [b,c]	92.9	2.2 [d]
Western	13.8	86.2	6.3	0.16 [b]	54.2	3.5 [c,d]	93.6	1.0 [a]	81.4	1.9 [a,b]	83.2	2.2 [b]	82.9	3.0 [b,c]	94.1	1.9 [d]
Isabel	3.5	96.6	6.3	0.15 [b]	49.2	5.3 [b,c,d]	99.3	0.5 [a,b]	91.5	1.7 [b]	84.6	4.0 [a,b,c,d]	73.6	4.3 [a,b,c]	90.2	3.6 [c,d]
Central	6.3	93.7	6.1	0.20 [a,b]	39.9	2.3 [b]	97.0	1.3 [a,b]	87.8	4.3 [a,b]	94.1	1.2 [d]	73.1	4.4 [a,b]	69.0	3.5 [b,c]
Rennel-Bellona	0.0	100.0	5.3	0.07	16.1	4.1 [a]	99.6	0.3 [b]	81.1	4.7 [a,b]	98.7	0.3	65.1	3.7 [a]	41.6	9.8 [a,b]
Guadalcanal	17.5	82.5	6.7	0.19 [b,c]	45.0	3.4 [b,c,d]	93.5	2.1 [a]	76.0	4.1 [a]	83.7	3.0 [b,c]	85.1	3.9 [a,b,c]	83.4	3.9 [c,d]
Malaita	3.6	96.4	6.5	0.17 [b,c]	54.6	5.3 [b,c,d]	98.0	0.8 [a,b]	80.3	4.5 [a,b]	90.0	3.4 [b,c,d]	89.6	2.7 [b,c]	82.2	2.7 [c,d]
Makira	4.8	95.2	6.2	0.11 [b]	32.3	5.2 [a,b,c]	98.5	1.3 [a,b]	85.5	2.8 [a,b]	94.5	0.9 [d]	88.9	2.3 [c]	45.2	6.0 [a]
Temotu	7.8	92.2	7.0	0.11 [c]	59.9	3.6 [d]	98.2	1.3 [a,b]	95.7	2.8 [a,b]	94.2	1.5 [c,d]	85.7	3.9 [a,b,c]	73.3	3.7 [b,c]
Honiara	100.0	0.0	5.6	0.06 [a]	3.4	0.7	38.1	2.1	7.6	1.3	67.1	2.1 [a]	97.2	0.7	99.7	0.2
**Wealth group**																
Lowest	2.3	97.7	6.0	0.10 [a]	36.7	2.6 [a]	97.4	0.9 [a]	84.6	2.5 [b]	88.5	2.0 [a,b]	80.1	2.0 [a]	62.2	3.6
2	7.4	92.6	6.3	0.09 [a,b]	48.5	3.1 [b,c]	96.3	0.7 [a]	84.0	1.9 [b]	88.3	1.6 [b]	84.7	2.0 [a,b,c]	78.0	2.6
3	14.9	85.1	6.4	0.11 [a,b]	51.7	3.3 [c]	94.1	1.0 [a]	76.9	2.6 [a,b]	85.7	2.7 [a,b]	86.3	1.8 [b]	88.5	1.9 [a]
4	24.8	75.3	6.6	0.09 [b]	48.0	3.2 [b,c]	88.4	1.5	71.5	2.9 [a]	87.2	1.8 [a,b]	87.0	2.7 [a,b]	91.7	1.6 [a]
Highest	39.5	60.5	6.5	0.11 [a,b]	39.4	2.9 [a,b]	76.1	2.3	56.9	3.5	81.2	1.8 [a]	91.9	1.7 [c]	92.3	1.6 [a]

FE, food environment. HH, household. Means or proportions sharing a superscript letter within a column and group (urban vs rural, province, wealth group) are not significantly different from each other at P<0.05

Supplementary Table S2: Proportion of total quantity of food acquired from different food environments Nationally, by geographic location and wealth groups.

					Provinces										Wealth group				
		National	Urban	Rural	Choiseul	Western	Isabel	Central	Rennel.	Guadal.	Malaita	Makira	Temotu	Honiara	1 (lowest)	2	3	4	5 (highest)
Cultivated	Gardens and subsistence	52.1	17.6	55.7	54.7	50.6	63.2	62.2	48.1	51.9	56.4	56.4	37.8	8.3	59.1	57.4	55.7	50.5	39.8
	Plantations and commercial	7.8	0.6	8.5	8.1	6.5	5.9	4.8	16.5	9.3	7.4	13.9	9.4	0.1	10.1	8.8	7.9	7.0	5.5
	**Cultivated - total**	59.9	18.2	64.2	62.8	57.1	69.1	67.0	64.6	61.2	63.7	70.3	47.2	8.4	69.3	66.2	63.6	57.5	45.3
Wild	Bush and forests	4.7	0.6	5.2	1.9	2.1	2.2	4.4	8.0	5.8	5.1	4.2	18.5	0.2	5.9	4.8	4.0	4.8	4.0
	Sea and reefs	8.0	1.2	8.7	9.2	13.3	11.4	10.9	6.8	3.4	7.1	7.1	11.2	0.1	6.8	8.5	8.7	9.1	7.0
	Rivers, lakes and streams	0.7	0.1	0.8	0.6	0.2	0.4	0.1	7.4	2.1	0.3	0.9	0.4	0.0	1.5	0.5	0.4	0.5	0.7
	Estuaries and mangroves	1.3	0.1	1.4	0.9	0.9	3.4	1.1	0.2	0.0	2.6	0.3	0.9	0.0	0.7	1.3	1.5	1.5	1.6
	**Wild - total**	14.8	1.9	16.1	12.7	16.6	17.4	16.5	22.3	11.3	15.1	12.4	31.1	0.3	14.8	15.1	14.6	15.8	13.3
Kin and community	Family and community	8.5	7.7	8.5	10.4	8.5	5.7	7.8	10.3	7.8	7.8	11.4	12.1	7.0	8.1	8.1	7.7	7.7	10.1
	Social and cultural gatherings	0.2	0.1	0.2	0.3	0.1	0.2	0.2	0.1	0.1	0.3	0.3	0.3	0.0	0.2	0.2	0.3	0.3	0.1
	**Kin and community - total**	8.7	7.8	8.8	10.8	8.6	6.0	8.0	10.3	7.9	8.1	11.7	12.3	7.0	8.3	8.3	8.0	8.0	10.2
Retail - informal	Canteen	2.5	6.8	2.1	1.9	1.5	1.9	1.0	0.8	3.9	2.1	2.1	2.2	8.0	1.6	1.9	2.4	2.6	3.8
	Opportunistic/mobile vendor	1.0	1.8	1.0	0.7	1.4	0.3	0.5	0.2	0.8	1.1	0.5	2.1	2.4	0.8	0.9	1.0	1.1	1.4
	Local market	4.2	18.5	2.7	4.3	5.5	0.8	2.3	0.1	3.5	4.4	1.4	2.0	17.0	2.2	2.4	3.2	5.2	7.2
	**Informal retail - total**	7.7	27.1	5.7	6.9	8.4	3.0	3.8	1.2	8.2	7.6	4.0	6.3	27.4	4.6	5.2	6.6	8.9	12.3
Retail - formal	Central market	2.6	19.7	0.8	0.5	0.9	0.0	0.1	0.0	3.9	1.2	0.0	0.6	28.8	0.4	0.8	1.6	2.8	7.3
	Store or shop	5.4	22.8	3.6	5.5	6.6	4.2	2.0	1.4	6.9	3.6	1.0	2.0	25.9	2.1	3.6	4.7	6.1	10.0
	Co-operative	0.0	0.1	0.0	0.0	0.0	0.2	0.3	0.0	0.0	0.0	0.0	0.0	0.0	0.0	0.0	0.0	0.1	0.0
	Restaurant	0.1	1.1	0.0	0.0	0.0	0.0	0.0	0.0	0.4	0.0	0.0	0.0	1.4	0.0	0.0	0.0	0.1	0.4
	Supermarket	0.0	0.4	0.0	0.0	0.0	0.0	0.0	0.0	0.1	0.0	0.0	0.0	0.6	0.0	0.0	0.0	0.0	0.1
	**Formal retail - total**	8.2	44.1	4.5	6.0	7.6	4.4	2.4	1.4	11.2	4.8	1.0	2.6	56.8	2.5	4.4	6.4	9.1	17.9
	Undetermined	0.8	0.8	0.8	0.9	1.8	0.1	2.3	0.2	0.2	0.6	0.6	0.6	0.2	0.6	0.7	0.9	0.7	1.0

Rennel., Rennel Belonna. Guadal., Guadalcanal.

Supplementary Table S3: Proportion of total value of food acquired from different food environments Nationally, by geographic location and wealth groups.

					Provinces										Wealth group				
		National	Urban	Rural	Choiseul	Western	Isabel	Central	Rennel.	Guadal.	Malaita	Makira	Temotu	Honiara	1 (lowest)	2	3	4	5 (highest)
Cultivated	Gardens and subsistence	31.0	8.0	37.0	30.8	29.7	38.6	38.5	26.0	28.0	42.9	35.6	24.3	3.8	42.32	38.3	36.56	31.6	20.15
	Plantations and commercial	2.0	0.5	2.4	3.2	1.7	1.7	1.4	3.8	2.3	1.9	5.0	3.2	0.1	2.9	2.38	2.05	1.78	1.36
	**Cultivated - total**	33.0	8.4	39.4	33.9	31.4	40.4	39.9	29.8	30.2	44.7	40.6	27.6	4.0	45.22	40.68	38.61	33.38	21.52
Wild	Bush and forests	2.8	0.3	3.4	1.3	1.5	2.5	3.2	5.8	2.8	2.0	3.4	18.6	0.1	4.09	2.95	2.21	2.8	1.99
	Sea and reefs	7.3	1.2	8.8	9.3	11.4	12.0	13.5	7.3	3.5	7.6	7.1	10.2	0.2	7.95	8.71	8.27	7.51	5.94
	Rivers, lakes and streams	0.5	0.1	0.6	0.6	0.3	0.5	0.1	2.2	1.1	0.3	1.0	0.3	0.0	0.88	0.53	0.41	0.39	0.33
	Estuaries and mangroves	1.2	0.0	1.4	0.8	0.7	3.1	1.1	0.1	0.0	2.7	0.3	0.7	0.0	0.5	1.05	1.26	1.6	1.09
	**Wild - total**	11.7	1.7	14.3	11.9	13.9	18.0	17.9	15.3	7.4	12.6	11.8	29.7	0.3	13.42	13.25	12.16	12.3	9.35
Kin and community	Family and community	11.9	7.0	13.2	14.2	11.5	9.7	14.0	31.5	11.2	12.0	19.6	16.6	5.8	15.65	13.08	11.74	10.33	11.27
	Social and cultural gatherings	0.3	0.2	0.3	0.3	0.5	0.2	0.2	0.1	0.3	0.4	0.3	0.4	0.1	0.33	0.26	0.48	0.38	0.21
	**Kin and community - total**	12.2	7.2	13.5	14.5	12.0	9.9	14.2	31.6	11.5	12.4	19.8	17.0	5.9	15.98	13.34	12.22	10.71	11.47
Retail - informal	Canteen	8.6	10.9	8.0	7.8	5.4	8.7	4.7	8.1	12.5	6.5	13.6	6.7	11.5	7.75	7.94	8.51	8.59	9.22
	Opportunistic/mobile vendor	1.2	1.7	1.1	0.8	1.5	0.5	0.5	0.3	1.1	1.0	1.1	2.4	2.2	0.99	1.28	1.18	1.25	1.39
	Local market	4.1	10.2	2.6	4.1	4.6	0.9	2.2	0.1	4.1	3.6	2.8	2.5	9.1	2.56	2.64	3.25	4.61	5.62
	**Informal retail - total**	14.0	22.8	11.7	12.7	11.5	10.1	7.4	8.5	17.7	11.2	17.5	11.5	22.8	11.29	11.86	12.94	14.45	16.22
Retail - formal	Central market	3.4	11.1	1.4	0.6	0.8	0.0	0.1	0.0	4.8	2.0	0.0	0.7	14.4	0.84	1.1	1.99	3.39	6.19
	Store or shop	22.0	40.1	17.3	24.5	26.8	20.3	10.6	13.4	25.2	15.5	8.2	11.5	42.1	11.18	16.97	19.59	22.68	29.16
	Co-operative	0.2	0.1	0.2	0.0	0.2	0.9	1.8	0.0	0.0	0.0	0.2	0.0	0.0	0.24	0.23	0.1	0.3	0.12
	Restaurant	0.8	3.2	0.2	0.0	0.1	0.0	0.1	0.0	1.1	0.0	0.0	0.1	4.6	0.051	0.068	0.15	0.35	2.01
	Supermarket	0.3	1.4	0.0	0.0	0.0	0.0	0.0	0.0	0.3	0.0	0.0	0.0	2.1	0.0053	0.083	0.036	0.13	0.77
	**Formal retail - total**	26.6	55.9	19.1	25.2	27.9	21.2	12.6	13.4	31.3	17.5	8.4	12.3	63.1	12.31	18.46	21.88	26.85	38.25
	Undetermined	2.5	4.0	2.1	1.9	3.4	0.5	8.1	1.4	1.8	1.7	1.9	2.0	3.9	1.79	2.41	2.19	2.3	3.18

Rennel., Rennel Belonna. Guadal., Guadalcanal.

Supplementary Table S4: Proportion of food groups (quantity) acquired from different food environments in Solomon Islands nationally.

		Breads and cereals	Roots and tubers	Vegetables	Fruits	Nuts	Fish and seafood	Meat	Eggs and dairy	Oils and fats	Discretionary food	Other
Cultivated	Gardens and subsistence	0.5	81.4	62.4	37.6	28.8	0.5	9.5	25.2	0.8	7.5	0.8
	Plantations and commercial	0.0	0.5	0.6	35.8	11.7	0.2	1.4	7.6	0.0	0.2	1.9
	**Cultivated - total**	0.5	81.8	63.0	73.4	40.5	0.7	11.0	32.8	0.8	7.7	2.7
Wild	Bush and forests	0.1	2.9	8.1	9.9	41.7	2.7	9.1	5.3	0.1	0.2	0.2
	Sea and reefs	0.0	0.1	1.6	0.8	0.1	57.7	0.0	0.2	0.0	0.0	0.0
	Rivers, lakes and streams	0.0	0.1	1.1	0.0	0.0	4.1	0.0	0.0	0.0	0.1	0.0
	Estuaries and mangroves	0.0	0.5	0.4	0.2	0.0	7.5	0.0	0.0	0.0	0.0	0.0
	**Wild - total**	0.2	3.6	11.2	10.9	41.8	72.0	9.1	5.5	0.1	0.3	0.2
Kin and community	Family and community	17.2	7.5	5.8	5.6	7.7	12.2	27.9	12.1	9.4	14.0	8.3
	Social and cultural gatherings	0.3	0.2	0.1	0.2	0.0	0.2	1.0	0.1	0.3	0.4	0.3
	**Kin and community - total**	17.5	7.7	5.9	5.7	7.7	12.4	28.9	12.2	9.7	14.4	8.5
Retail - informal	Canteen	21.7	0.2	0.4	0.3	0.6	2.2	1.6	2.6	21.8	21.5	23.2
	Opportunistic/mobile vendor	0.9	0.7	1.2	0.8	0.7	1.7	1.9	1.7	2.5	5.3	0.8
	Local market	1.0	3.6	9.1	4.1	5.8	4.5	2.7	6.6	3.0	6.2	0.8
	**Informal retail - total**	23.6	4.4	10.7	5.2	7.1	8.4	6.1	10.9	27.2	33.0	24.8
Retail - formal	Central market	0.2	1.7	7.4	3.8	1.8	2.8	2.0	4.5	0.6	1.7	0.4
	Store or shop	54.9	0.2	1.2	0.3	0.3	2.8	35.8	27.8	58.0	38.0	49.8
	Co-operative	0.3	0.0	0.0	0.0	0.0	0.0	0.1	0.2	0.7	0.3	0.3
	Restaurant	0.5	0.0	0.0	0.0	0.0	0.0	2.3	1.9	0.6	2.4	3.9
	Supermarket	0.2	0.0	0.0	0.0	0.0	0.0	2.9	1.0	0.2	0.2	0.3
	**Formal retail - total**	56.0	1.9	8.6	4.2	2.1	5.7	43.0	35.4	60.0	42.6	54.6
	Undetermined	2.1	0.5	0.7	0.6	0.7	0.9	1.9	3.2	2.2	2.1	9.1

Supplementary Table S5: Proportion of food groups (value) acquired from different food environments in Solomon Islands nationally.

		Breads and cereals	Roots and tubers	Vegetables	Fruits	Nuts	Fish and seafood	Meat	Eggs and dairy	Oils and fats	Discretionary food	Other
Cultivated	Gardens and subsistence	0.5	78.7	57.0	50.7	33.7	1.0	24.7	12.5	0.6	1.6	0.9
	Plantations and commercial	0.0	0.5	0.6	19.7	6.7	0.1	4.0	10.1	0.0	0.1	1.7
	**Cultivated - total**	0.5	79.2	57.7	70.3	40.4	1.1	28.6	22.5	0.6	1.6	2.7
Wild	Bush and forests	0.1	3.7	6.5	5.5	33.4	1.9	6.6	1.8	0.0	0.2	0.2
	Sea and reefs	0.0	0.2	3.1	1.1	0.1	38.9	0.0	0.1	0.0	0.0	0.0
	Rivers, lakes and streams	0.0	0.2	0.9	0.0	0.0	2.0	0.0	0.0	0.0	0.0	0.0
	Estuaries and mangroves	0.0	0.5	0.7	0.6	0.0	5.2	0.0	0.0	0.0	0.0	0.0
	**Wild - total**	0.1	4.5	11.2	7.2	33.5	48.0	6.6	1.9	0.0	0.2	0.2
Kin and community	Family and community	17.1	8.4	5.8	7.2	8.4	13.9	23.0	9.0	8.4	12.6	8.3
	Social and cultural gatherings	0.3	0.3	0.1	0.2	0.0	0.4	0.6	0.2	0.3	0.4	0.7
	**Kin and community - total**	17.4	8.6	5.9	7.3	8.4	14.3	23.6	9.3	8.6	13.0	9.0
Retail - informal	Canteen	17.8	0.2	0.8	0.3	1.3	8.0	1.1	3.7	19.3	24.9	12.8
	Opportunistic/mobile vendor	0.6	0.7	1.2	0.9	0.8	1.5	2.0	1.0	1.8	3.0	1.4
	Local market	0.8	4.0	9.9	5.7	7.0	6.0	2.2	3.1	2.7	3.7	1.6
	**Informal retail - total**	19.2	4.9	11.9	6.9	9.1	15.5	5.3	7.8	23.7	31.6	15.9
Retail - formal	Central market	0.2	2.1	9.9	6.4	4.7	7.4	1.2	3.7	0.4	0.9	0.5
	Store or shop	58.7	0.2	2.6	0.7	3.0	12.3	26.8	44.6	63.1	47.6	25.1
	Co-operative	0.5	0.0	0.0	0.0	0.1	0.1	0.0	0.3	0.9	0.5	0.2
	Restaurant	0.5	0.0	0.1	0.1	0.1	0.1	1.7	1.4	0.8	1.9	10.5
	Supermarket	0.3	0.0	0.1	0.1	0.0	0.0	2.8	2.1	0.2	0.6	0.3
	**Formal retail - total**	60.2	2.3	12.7	7.2	7.8	19.9	32.5	52.1	65.5	51.4	36.6
	Undetermined	2.5	0.5	0.6	1.0	0.9	1.2	3.4	6.4	1.6	2.2	35.7

Supplementary Table S6: Effect of reliance on different FEs (independent variables) on two measures of diet quality (fruit and vegetable acquisition, and ultra-processed food acquisition), full model including potential confounders.

	Outcome variable: fruit and vegetable acquisition (g/AME/day)						
**Independent variables**	β coefficient	SE	t	P value	P value for log-transformed model	CI lower bound	CI upper bound
Reliance on formal retail FE (binary)	-178.84	50.10	-3.57	0.001	0.016	-278.14	-79.55
Reliance on informal retail FE (binary)	-37.65	52.32	-0.72	0.473	0.270	-141.34	66.04
Reliance on cultivated FE (binary)	118.70	36.17	3.28	0.001	0.002	47.02	190.38
Reliance on wild FE (binary)	239.08	40.22	5.94	<0.001	<0.001	159.36	318.81
Reliance on kin and community FE (binary)	192.01	38.58	4.98	<0.001	<0.001	115.56	268.47
Rural (vs urban)	177.51	42.83	4.14	<0.001	0.282	92.62	262.40
Female household head (vs male)	111.35	36.25	3.07	0.003	0.002	39.51	183.20
Expenditure quintile (poorest)							
2	58.75	37.01	1.59	0.115	0.136	-14.61	132.11
3	130.21	42.15	3.09	0.003	0.005	46.67	213.75
4	201.79	60.78	3.32	0.001	<0.001	81.31	322.26
5 (richest)	240.37	65.71	3.66	<0.001	0.001	110.14	370.59
Education level of household head (no school)							
Primary	-31.03	30.98	-1.00	0.319	0.656	-92.43	30.37
Secondary +	-26.87	27.34	-0.98	0.328	0.946	-81.05	27.31
Age of household head (years)	-4.62	1.25	-3.69	<0.001	0.063	-7.11	-2.14
HH size (number of members)	-33.55	5.75	-5.83	<0.001	<0.001	-44.94	-22.15
	Outcome variable: UPFs acquisition (g/AME/day)						
Reliance on formal retail FE (binary)	8.04	1.41	5.69	<0.001	<0.001	5.24	10.84
Reliance on informal retail FE (binary)	5.47	1.45	3.78	<0.001	0.001	2.61	8.34
Reliance on cultivated FE (binary)	-2.68	2.63	-1.02	0.310	0.355	-7.90	2.54
Reliance on wild FE (binary)	-3.59	1.89	-1.90	0.060	0.008	-7.33	0.16
Reliance on kin and community FE (binary)	0.40	2.09	0.19	0.847	0.263	-3.74	4.55
Rural (vs urban)	-21.01	2.88	-7.30	0.000	<0.001	-26.72	-15.31
Female household head (vs male)	0.39	2.07	0.19	0.851	0.891	-3.71	4.49
Expenditure quintile (poorest)							
2	4.02	1.02	3.95	<0.001	<0.001	2.00	6.04
3	9.45	1.57	6.03	<0.001	<0.001	6.35	12.55
4	14.75	1.64	9.00	<0.001	<0.001	11.50	18.00
5 (richest)	29.51	2.12	13.94	<0.001	<0.001	25.31	33.70
Education level of household head (no school)							
Primary	2.02	1.15	1.76	0.081	0.542	-0.26	4.30
Secondary +	3.41	1.16	2.93	0.004	0.025	1.10	5.71
Age of household head (years)	-0.16	0.05	-3.27	0.001	<0.001	-0.25	-0.06
HH size (number of members)	-1.74	0.26	-6.75	<0.001	<0.001	-2.25	-1.23

Shaded cells indicates where use of the log-transformed model P-value would result in different interpretation of results.
